# Human immune response against salivary antigens of *Simulium damnosum* s.l.: A new epidemiological marker for exposure to blackfly bites in onchocerciasis endemic areas

**DOI:** 10.1371/journal.pntd.0009512

**Published:** 2021-06-22

**Authors:** Laura Willen, Maria-Gloria Basáñez, Vit Dvorak, Francis B. D. Veriegh, Frank T. Aboagye, Bright Idun, Maha Elhadi Osman, Mike Y. Osei-Atweneboana, Orin Courtenay, Petr Volf

**Affiliations:** 1 Department of Parasitology, Faculty of Science, Charles University, Prague, Czech Republic; 2 MRC Centre for Global Infectious Disease Analysis and London Centre for Neglected Tropical Disease Research, Department of Infectious Disease Epidemiology, School of Public Health, Imperial College London, London, United Kingdom; 3 Biomedical and Public Health Research Unit, CSIR-Water Research Institute, Accra, Ghana; 4 Commission for Biotechnology and Genetic Engineering, National Centre for Research, Khartoum, Sudan; 5 Zeeman Institute for Systems Biology & Infectious Disease Epidemiology Research and School of Life Sciences, University of Warwick, Coventry, United Kingdom; University of Buea, CAMEROON

## Abstract

**Background:**

*Simulium damnosum* sensu lato (s.l.) blackflies transmit *Onchocerca volvulus*, a filarial nematode that causes human onchocerciasis. Human landing catches (HLCs) is currently the sole method used to estimate blackfly biting rates but is labour-intensive and questionable on ethical grounds. A potential alternative is to measure host antibodies to vector saliva deposited during bloodfeeding. In this study, immunoassays to quantify human antibody responses to *S*. *damnosum* s.l. saliva were developed, and the salivary proteome of *S*. *damnosum* s.l. was investigated.

**Methodology/Principal findings:**

Blood samples from people living in onchocerciasis-endemic areas in Ghana were collected during the wet season; samples from people living in Accra, a blackfly-free area, were considered negative controls and compared to samples from blackfly-free locations in Sudan. Blackflies were collected by HLCs and dissected to extract their salivary glands. An ELISA measuring anti-*S*. *damnosum* s.l. salivary IgG and IgM was optimized and used to quantify the humoral immune response of 958 individuals. Both immunoassays differentiated negative controls from endemic participants. Salivary proteins were separated by gel-electrophoresis, and antigenic proteins visualized by immunoblot. Liquid chromatography mass spectrometry (LC–MS/MS) was performed to characterize the proteome of *S*. *damnosum* s.l. salivary glands. Several antigenic proteins were recognized, with the major ones located around 15 and 40 kDa. LC–MS/MS identified the presence of antigen 5-related protein, apyrase/nucleotidase, and hyaluronidase.

**Conclusions/Significance:**

This study validated for the first time human immunoassays that quantify humoral immune responses as potential markers of exposure to blackfly bites. These assays have the potential to facilitate understanding patterns of exposure as well as evaluating the impact of vector control on biting rates. Future studies need to investigate seasonal fluctuations of these antibody responses, potential cross-reactions with other bloodsucking arthropods, and thoroughly identify the most immunogenic proteins.

## Author summary

Measuring biting rates of bloodsucking arthropods has been proved to be a crucial tool in understanding transmission dynamics and evaluating control measures against vector-borne diseases. Blackflies of the *S*. *damnosum sensu lato* (s.l.) complex are the prime vectors of *Onchocerca volvulus*, a filarial nematode and causative agent of human onchocerciasis (or river blindness) in Africa. Blackfly biting rates are typically measured by human attractant vector collectors who capture host-seeking female flies as they alight on their skin but before they take a blood meal. Apart from the ethical concerns raised by this method, estimates thus obtained do not reflect true exposure to vector bites. An understanding of whether there are people who receive more bites than others and who may be at higher risk of acquiring the infection is crucial to modelling control interventions. Therefore, in the present study we developed IgG- and IgM-based immunoassays to quantify antibody responses in human populations to salivary antigens of *S*. *damnosum* s.l. in onchocerciasis-endemic communities of Ghana. The proteome of *S*. *damnosum* s.l. salivary glands was also investigated to potentially identify salivary antigens that could be employed as exposure markers against this important vector group.

## Introduction

Human onchocerciasis or river blindness is a severely debilitating parasitic disease caused by the filarial nematode *Onchocerca volvulus* (Nematoda: Filarioidea) and is transmitted via bites of infective haematophagous female blackflies (Diptera: Simuliidae). Although the disease has been endemic in six Latin-American countries and still occurs in Yemen, 99% of the cases are found in Africa where the most important vectors belong to the *Simulium damnosum sensu lato* (s.l.) species complex [1]. The sibling species within this complex vary in their ecology and geographical distribution, and exhibit different degrees of anthropophagy, vector competence, and vectorial capacity [2, 3].

The World Health Organization’s 2021–2030 Roadmap for Neglected Tropical Diseases has proposed that elimination (interruption) of transmission of onchocerciasis be verified in an increasing number of countries by 2030 [4]. Transmission dynamics models helped to inform the formulation of this roadmap, and indicated that vector control (where possible, and in addition to regular treatment of human populations) would be favourable in reducing blackfly biting rates, and hence transmission, in highly endemic areas [5]. An important determinant of the feasibility of elimination is the baseline (pre-intervention) level of endemicity, defined by the initial infection prevalence. Onchocerciasis prevalence exhibits a strongly non-linear relationship with the annual biting rate (ABR, the number of blackfly bites per person per year) [6], such that high prevalence values can be associated with a broad range of ABRs [7]. Crucial modelling uncertainties include the assumed degree of exposure heterogeneity in the human population, and the age- and sex-specific patterns of exposure [7, 8]. Stronger heterogeneity (ultimately resulting in few hosts harbouring most parasites) hinders elimination [9]. However, measuring ABRs is labourious, time-consuming and mostly reliant on human landing catches (HLCs) [10]. Measuring individual heterogeneity in exposure to blackfly bites requires counting the actual number of blackflies landing and successfully feeding on individual villagers during their routine day-time activities [11]. These procedures raise ethical concerns about enhanced risk of infection for those involved and are unable to provide robust measures of effective exposure to biting between and within endemic communities. Therefore, developing and validating alternative methods for measuring exposure to blackfly bites is highly desirable. Data obtained using such methods would improve the parameterization of onchocerciasis transmission models by using independent exposure data, since currently exposure patterns are inferred from parasitological data, which compound exposure to vector bites and infection processes [7]. At the same time, such methods would provide invaluable tools to evaluate and compare the impact of various anti-vectorial approaches for onchocerciasis control and elimination [12–14]. Developing assays to quantify anti-blackfly’s saliva antibody responses in endemic human populations would potentially provide such tools, as demonstrated with sand flies and leishmaniasis [15, 16].

Saliva of haematophagous arthropods is known to contain a vast array of pharmacologically-active compounds with anti-haemostatic (i.e. vasodilators, inhibitors of blood coagulation and platelet aggregation), anti-inflammatory and immunomodulatory activities (reviewed in [17]). Besides the pharmacological function of many of these compounds, studies on mosquitoes, triatomines, tsetse flies, and sand flies have proved that their salivary proteins provide useful tools to develop immunoassays to estimate host exposure to their bites [16, 18–21]. In blackflies, salivary components of relatively few species have been researched; in fact, immunomodulatory activities and (murine) humoral immune responses against salivary antigens have only been identified for *S*. *vittatum* [22, 23]. The saliva injected when blackflies bite causes a pronounced and persistent erythema, together with intense itching and local swelling. Previous studies have demonstrated the occurrence of IgE-mediated allergic dermatitis in horses induced by *S*. *vittatum* saliva [24] and IgE-mediated hypersensitivity in humans who exhibited allergic reactions to the bite of *S*. *nigrogilvum* [25]. However, no assays have been developed to measure human exposure to simuliid vectors of human onchocerciasis.

In this study, we examine the humoral immune response against *S*. *damnosum* s.l. in humans living in onchocerciasis-endemic communities in Ghana and propose a novel tool to evaluate blackfly exposure in onchocerciasis epidemiological and vector control studies. Ghana was one of the first countries to initiate vector control activities by large-scale aerial larviciding of blackfly breeding sites under the former Onchocerciasis Control Programme in West Africa (OCP, 1974–2002) [26]. It was also one of the first countries to commence mass drug administration (MDA) to complement vector control after the completion of a 5-year community trial of ivermectin (Mectizan), conducted in the former hyperendemic focus of Asubende [27]. Currently, ivermectin MDA is the mainstay of onchocerciasis control and elimination in Africa (including Ghana) as large-scale deployment of blackfly control is no longer performed. The focus of Asubende has been the subject of comprehensive entomological research in relation to onchocerciasis transmission [28–31], and became a source of epidemiological and entomological data for parameterization of transmission models [3, 32, 33]. In this paper, we report on the development of immunoassays against *S*. *damnosum* s.l. salivary antigens, provide a characterization of its proteome, and discuss the potential use of certain salivary components as host exposure markers, the results of which can be used to improve our understanding of onchocerciasis transmission dynamics towards its control and elimination in African settings.

## Materials and methods

### Ethics statement

Ethical clearance was obtained from the Council for Scientific and Industrial Research (CSIR) Institutional Review Board (RPN008/CSIR-IRB/2019). Prior to the study, all participants were informed in detail in their local language about the purpose of the study. Fully informed signed consent was subsequently obtained from the participant, or from the parent/ guardian in case the participant was under 18 years of age. All participants were registered before blood sampling. Data were anonymized. Participating individuals were not at an increased risk of exposure. All participants were living in an area that is part of the national MDA programme through which they receive biannual ivermectin treatment [34, 35]. Blackflies were collected by OCP vector collectors following standard OCP vector collector techniques [10]. Archived Sudanese human plasma samples used to validate the assays presented in this paper were obtained under ethical approval as previously described [36].

### Blood samples

Blood samples of 958 people living in rural villages in an onchocerciasis endemic area in the Bono East region (a new region, created from partitioning the former, larger Brong-Ahafo region threefold) of Ghana were collected during the wet season in August 2019. The area is reported to have high *S*. *damnosum* s.l. biting rates as determined by standard OCP vector collector techniques [29, 37]. Blood samples were obtained from village residents over the age of 5 years, with a target of at least 100 per age/sex class (age categories in years: 5–10, 11–20, 21–30, 31–40, 41–50, 51–60, 61–70, >71) by adopting two approaches depending on the population size of the village. In villages where the numbers within age/sex categories were generally small, all participants were offered the possibility of recruitment; in larger villages an age/sex stratified sample was identified based on inspection of the population medical register of MDA treatment contained in three books, in which the family names of all villagers were recorded. Within-book page numbers were selected by a random number generator in R software, and the corresponding families were invited to participate. This procedure was repeated until the target numbers within age/sex classes was reached in equal numbers as far as logistically possible. Blood samples from the OCP vector collectors living in the same area and expected to have experienced high biting rates were also collected and served as positive control samples during the preparation of the immunoassays. Thirty-five people at the Water Research Institute (CSIR) in Accra aged between 30 and 59 years of age and no known history of travelling to blackfly/ onchocerciasis-endemic areas were used as non-simuliid bitten negative controls. Twenty samples from people living in blackfly-free rural communities in Gedaref state and the city of Khartoum, Sudan, were used to further validate the negative control plasma samples from Accra. From each recruit 2 ml of blood was collected, placed in a cool box for transport, and spun down at 2,000 × g for 15 min to transfer the plasma to a separate tube which was stored at –20° C until testing.

### Blackfly sample collection and identification

In Bono East, blackfly biting rates have previously been recorded in the dry season (2,061 bites/person/month in Asubende; 775 in Agborlekame [29]; 500–2,500 bites/person/month in Asubende/Senyase and Aborlekame/Tainso [37], with only *S*. *damnosum sensu stricto* (s.s.)/*S*. *sirbanum* (the savannah members of the *S*. *damnosum* s.l. complex) being identified along the Pru river [28, 29]. In other regions of Ghana where dry and wet season sampling has been conducted, biting rates were higher in the wet season [29]. Therefore, for this study, sampling was performed in the wet season, the period with anticipated higher biting rates and plausibly also higher antibody responses.

Host-seeking blackflies were collected along the Pru river near one of the study villages (Asubende; 08° 01’ 01.4" N, 00° 58’ 53.8" W) by two OCP vector collectors using standard HLC techniques [10]. Blackflies were collected for 15 consecutive days in August 2019, from 7 a.m. until 6 p.m. or until the daily target of 200 blackfly females was reached. Pairs of workers collected flies in hourly intervals alternating between themselves. Collectors sat with bare lower legs and arms to capture landing female blackflies individually in polypropylene tubes (7.5 cm × 1.2 cm, Sigma-Aldrich), and immediately on landing before the flies took a blood meal. The blackflies were placed in a cool box for transport until dissection later the same day.

A previous study conducted in the same area in Ghana demonstrated that all female flies caught by vector collectors were within the *S*. *damnosum* s.l. species complex [29]. To confirm this, a subset of the caught specimens (n = 18) were stored in 96% ethanol, their total genomic DNA extracted by High Pure PCR Template Preparation Kit (Roche Life Science, Penzberg, Germany), and a fragment of mitochondrial *ND4* gene amplified using the ND4-1/ ND4-2 primer pair using reaction conditions as described previously [38]. PCR products were purified using a QIAquick PCR Purification Kit (Qiagen, Hilden, Germany) and directly sequenced in both directions using the same amplification primers. The obtained nucleotide sequences were edited and aligned using BioEdit software (7.0.9.0) [39] and compared by BLAST (basic local alignment search tool) with those available in GenBank.

### Dissection of blackfly salivary glands and preparation of the antigen

A total of 940 blackfly salivary glands were extracted by dissection under a stereomicroscope, immediately after the flies were anesthetized by placing them in a –20° C freezer for 10 min. Salivary glands were dissected in aliquots of 20 glands, stored in Tris-buffered saline (TBS, pH 7.5) at a concentration of one salivary gland per μl TBS and kept in a cool box during dissections. The aliquots were stored at –20° C until preparation of the assays.

Prior to the immunoassay and western blot analyses, the salivary glands were disrupted by three quick freeze-and-thaw cycles in liquid nitrogen. The aliquots of the salivary glands homogenates (SGH) were then spun at 13,500 rpm for 5 min to pellet any debris, and the supernatant of all aliquots was collected and pooled together. The concentration of the final pool was approximated by a nanodrop ND-1000 spectrophotometer (ThermoFisher Scientific) at 280 nm and was ± 0.94–1.02 mg/ml. The pool of SGH was aliquoted and used for both the enzyme-linked immunosorbent assays (ELISA) and the western blot.

### Enzyme-linked immunosorbent assays

Optimal antigen concentration and plasma dilution to measure anti-*S*. *damnosum* s.l. IgG were identified during an optimization process of which details are provided in Text A in S1 File.

All 958 plasma samples were then tested by ELISA for IgG, and a subset of 500 samples selected by random number generation from the full sample set within stratified age-classes (target 65 per age-class) was tested for IgM, based on the optimal dilutions defined during the preliminary assays. For the IgG and IgM assays, 96-well microtiter plates were coated, respectively, with 0.2 μg and 0.025 μg of SGH in 100 μl of 20 mM carbonate-bicarbonate coating buffer (pH 9.5) per well. After overnight incubation at 4° C, the plates were washed twice with phosphate-buffered saline with 0.05% Tween 20 (PBS-Tw), and blocked with 6% non-fat dried milk (Bio-Rad) diluted in PBS-Tw for 1 h at 37° C. After washing twice with PBS-Tw, the plates were incubated with plasma diluted at 1/100 (IgG) or 1/50 (IgM) in 2% non-fat dried milk and incubated for 1.5 h at 37° C. Plates were subsequently washed 6 times with PBS-Tw and incubated for 45 min at 37° C with peroxidase-conjugated anti-human IgG or IgM antibody (Sigma-Aldrich; Bethyl Laboratories, Inc.) diluted at 1/1,000 and 1/100,000 in PBS-Tw, respectively. The plates were washed 6 times and the chromogenic reaction was developed in the dark using an orthophenylendiamine (OPD) solution in a phosphate-citrate buffer (pH 5.5) with 0.1% hydrogen peroxide. The reaction was stopped after 5 min with 10% sulfuric acid and the absorbance (OD value) was measured at 492 nm using a Tecan Infinite M200 microplate reader (Schoeller). In each step, 100 μl of solution was used per well.

### Standardization

All samples were tested in duplicate. Samples with a coefficient of variation of more than 20% were retested. Each plate included a blank control well of which the OD was subtracted from the sample OD values for preliminary interrogation of the results. In order to correct for inter-plate variability, a set of positive control (PC) and negative control (NC) samples was included in each plate and standardized OD values (SOD) were generated according to the following formula: SOD = OD _sample_/(Average OD_PC_−average OD_NC_).

### Statistical analysis

All statistical analyses were performed using R software [40]. The median OD values between the optimization assays and the median SOD values between the different groups in the final assay were compared using the Wilcoxon signed rank sum test with Holm correction. A *P*-value of <0.05 was considered statistically significant. Cut-off values for the final assays were calculated based on the mean of the negative control samples plus three standard deviations. The results were graphically presented using the “ggplot2” package in R software [41].

### Sodium dodecyl sulphate–polyacrylamide gel electrophoresis (SDS-PAGE) and western blot

The salivary proteins present in blackfly SGH were electrophoretically separated on a 12% polyacrylamide gel under non-reducing conditions using a Mini-protean apparatus (Bio-Rad) (200 V, 55 min). A total of 4 μg of SGH was loaded per lane. One lane containing the blackfly separated salivary proteins was silver-stained, the rest was blotted onto the nitrocellulose membrane using the iBLOT instrument (Invitrogen), according to the manufacturer’s instructions. The nitrocellulose membrane was cut into strips and blocked at 4° C overnight in 6% milk diluted in TBS with 0.05% Tween 20 (TBS-Tw). The immunoblot was carried out with five negative control samples and 20 endemic plasma samples stratified by the IgG SOD values of <0.5 (group 1), 0.5–1.5 (group 2), and >1.5 (group 3). The nitrocellulose strips were then incubated with the individual samples diluted at 1:100 in TBS-Tw. After 1 h, all strips were washed with TBS-Tw and incubated for 1 h with anti-human IgG HRP conjugated antibody (1:1,000) (Sigma-Aldrich). Finally, the chromogenic reaction was developed with a substrate solution containing 3,3′-diaminobenzidine (Sigma-Aldrich) and H_2_O_2_.

### Protein characterization by liquid chromatography-tandem mass spectrometry

To characterize the proteins by liquid chromatography-tandem mass spectrometry (LC–MS/MS) analysis via in-gel digestion, blackfly SGH was electrophoretically separated following the same protocol as used for the Western Blot and stained with Coomassie G-250. Individual bands containing the proteins of interest were then excised from the Coomassie G-250 stained SDS-PAGE gel using sterile scalpels and cut into small pieces (approx. 1 mm × 1 mm). Bands were de-stained by sonication for 30 min in 50% acetonitrile (ACN) and 50 mM ammonium bicarbonate (ABC). After de-staining, the solution was removed and the gels were dried in ACN. Disulfide bonds were reduced using 10 mm dithiothreitol (DTT) in 100 mM ABC at 60° C for 30 min. Samples were again dried with ACN and free cysteine residues were blocked at room temperature in the dark for 10 min using 55 mM iodoacetamide in 100 mM ABC. Samples were dried thoroughly and the digestion buffer (10% ACN, 40 mM ABC and 13 ng/μl trypsin) was added to cover the gel pieces. Proteins were digested at 37° C overnight, after which they were sonicated for 30 min using 150 μl of 50% ACN with 0.5% formic acid. The supernatant containing the peptides was transferred to a new microcentrifuge tube to which 150 μl of elution solution was added. Following a 30 min sonication, the solution was removed, combined with the previous solution and dried using Speedvac. The dried peptides were reconstituted in 2% ACN with 0.1% trifluoroacetic acid (TFA) and injected into the Ultimate 3000 Nano LC coupled to the Orbitrap Fusion.

Nano Reversed phase column (EASY-Spray column, 50 cm × 75 μm ID, PepMap C18, 2 μm particles, 100 Å pore size) was used for LC–MS/MS analysis. Mobile phase buffer A was composed of water and 0.1% formic acid. Mobile phase B was composed of ACN and 0.1% formic acid. Samples were loaded onto the trap column (Acclaim PepMap300, C18, 5 μm, 300 Å Wide Pore, 300 μm × 5 mm) at a flow rate of 15 μl/min. The loading buffer was composed of water, 2% ACN and 0.1% TFA. Peptides were eluted with a gradient of B going from 4% to 35% over 60 min at a flow rate of 300 nl/min. The eluted peptide cations were converted to gas-phase ions by electrospray ionization and analyzed on a Thermo Orbitrap Fusion (Q-OT- qIT, Thermo). Survey scans of the peptide precursors from 350 to 1,400 m/z were performed at 120 K resolution (at 200 m/z) with a 5 × 10^5^ ion count target. Tandem MS was performed by isolation at 1.5 Th with the quadrupole, higher energy collisional dissociation (HCD) fragmentation with normalized collision energy of 30, and rapid scan MS analysis in the ion trap. The MS/MS ion count target was set to 10^4^ and the maximum injection time was 35 ms. Only those precursors with charge state 2–6 were sampled for MS/MS. The dynamic exclusion duration was set to 45 s with a 10 ppm tolerance around the selected precursor and its isotopes. The monoisotopic precursor selection was turned on. The instrument was run in top speed mode with 2 s cycles [42].

All data were analyzed and quantified with the MaxQuant software (version 1.6.1.0) [43]. The false discovery rate (FDR) was set to 1% for both proteins and peptides and a minimum length of seven amino acids was specified. The Andromeda search engine was used to screen the MS/MS spectra against the Simuliidae database (downloaded from NCBI in June 2020, containing 16,564 entries), and the *Culicoides nubeculosus* (downloaded from Uniprot in May 2020, containing 79 entries) and *Culicoides sonorensis* databases (downloaded from Uniprot in March 2021, containing 21,090 entries) (Ceratopogonidae). Enzyme specificity was set as C-terminal to Arg and Lys allowing cleavage at proline bonds and a maximum of two missed cleavages. Carbamydomethylation of cysteines was selected as a fixed modification and N-terminal protein acetylation, methionine oxidation and serine/threonine phosphorylation were selected as variable modifications. Data analysis was performed using Perseus 1.5.2.4 software [44].

## Results

### ELISA optimization

The optimal dilutions for the SGH, the anti-human IgG and IgM conjugates and the plasma samples were determined during preliminary assays. Details of the optimization process are provided in Text A and Figs A and B and Table A in S1 File.

### Human IgG and IgM immunological responses

**Fig 1:**
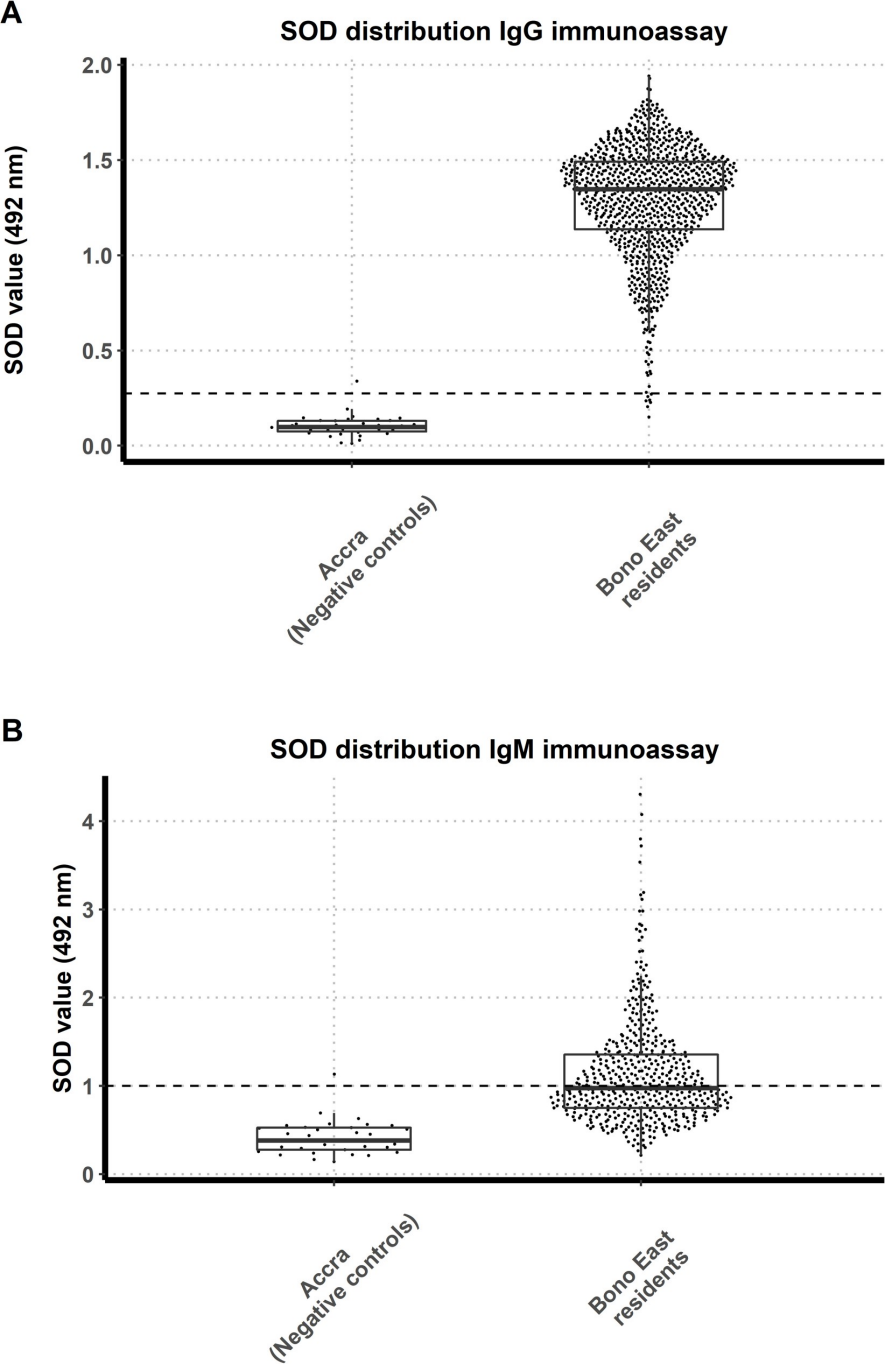
Distribution of standardized optical density (SOD) values for the IgG and IgM ELISA. **A.** SOD values of the IgG ELISA for negative control samples (n = 35) from Accra and Bono East region residents (n = 958), Ghana. The cut-off value is shown by a dashed horizontal line and is equal to 0.275 SOD. **B.** SOD values of the IgM ELISA for negative control samples (n = 33) and Bono East region residents (n = 500). The cut-off value is shown by a dashed horizontal line and is equal to 0.998 SOD. The solid black horizontal line within the boxes is the median; the lower and upper borders are, respectively, the 1st (Q1) and 3rd (Q3) quartiles; the vertical bars indicate the ‘minimum’ and ‘maximum’ values, calculated as Q1–1.5 × IQR (interquartile range) and Q1 + 1.5 × IQR, respectively.

All endemic Bono East human samples collected during the study (n = 958) and a subset of 500 samples were tested in duplicate by the IgG and IgM ELISA, respectively, together with negative control samples (n = 35 and n = 33 for the IgG and IgM ELISA, respectively). According to the cut-off values, 48.4% of the participants was positive for IgM, whereas 99.5% tested positive for IgG. The distributions of the SOD values for both ELISAs are shown in Fig 1. A statistically significant difference was detected between the median IgG SOD values of the Bono East test samples (median = 1.35 [quartile Q1: 1.14, quartile Q3: 1.49]) and the negative control plasma samples (median = 0.0987 [Q1: 0.08, Q3: 0.13]) (*P*<0.001). Median IgM SOD values for the two groups were also significantly different (median = 0.975 [Q1: 0.75, Q3: 1.36] and 0.384 [Q1: 0.28, Q3: 0.53]), respectively (*P*<0.001).

Lastly, 20 plasma samples from individuals living in blackfly-free rural communities in Gedaref state and the city of Khartoum, Sudan, were used to validate the control samples from Accra in the IgG ELISA. The distributions of the IgG SOD values for both negative controls groups were similar and not significantly different (Fig C in S1 File).

### Immunoblot with blackfly salivary proteins

**Fig 2:**
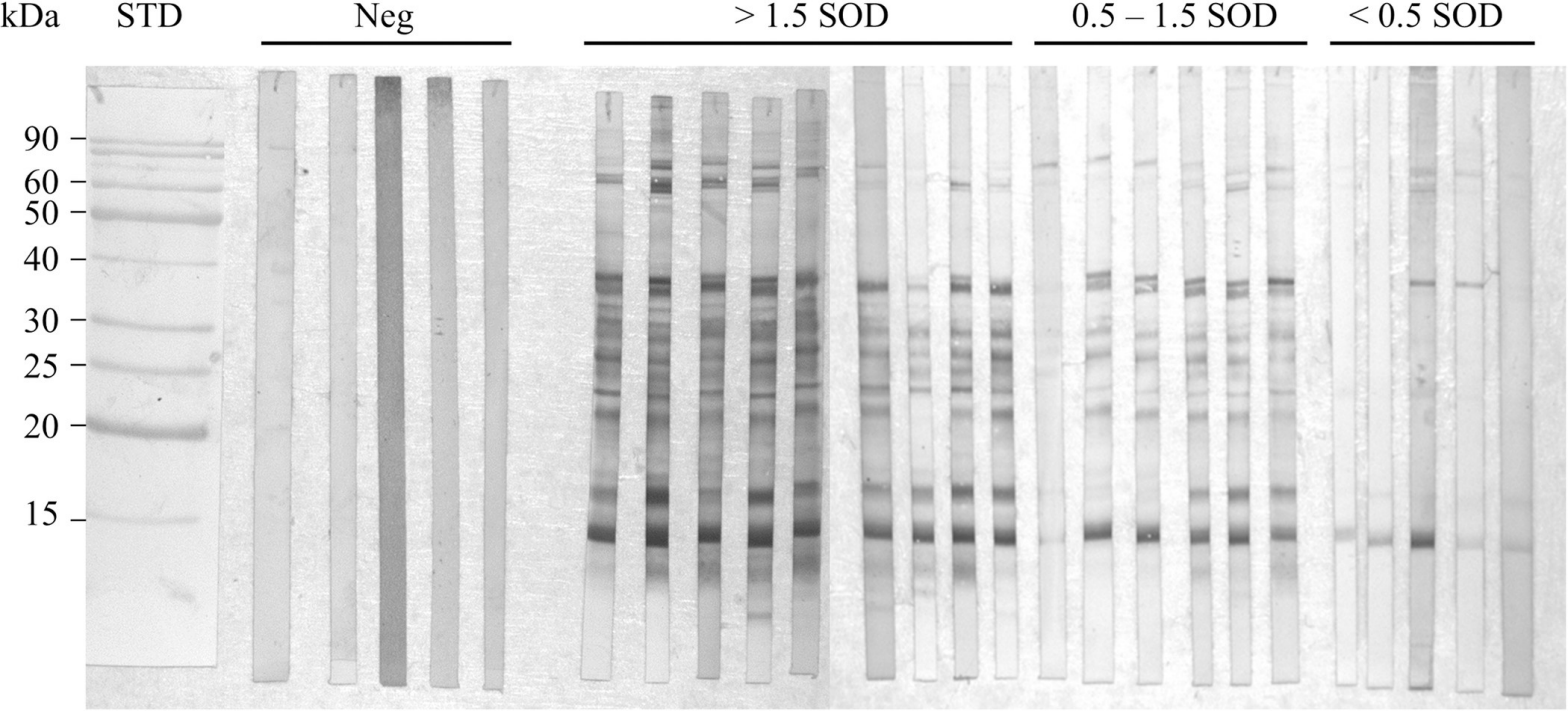
Immunoblot of salivary gland homogenate (SGH) of *Simulium damnosum sensu lato*. Five negative plasma samples from people living in Accra (Neg); nine plasma samples from people with an SOD value in the IgG ELISA greater than 1.5 (>1.5 SOD); six samples from people with an SOD value between 0.5 and 1.5 (0.5–1.5 SOD), and 5 samples from people with an SOD value lower than 0.5 (<0.5 SOD) were tested by western blot against SGH of *S*. *damnosum* s.l. STD: standard; SOD: Standardized Optical Density.

*Simulium damnosum* s.l. IgG immunogenic proteins were identified by immunoblotting (Fig 2). A total of 20 plasma samples of individuals from the Bono East region were tested together with five negative control samples from people living in Accra. The positive samples were chosen according to their SOD value in the anti-*S*. *damnosum* s.l. IgG ELISA, representing SOD values of <0.5 (group 1), 0.5–1.5 (group 2), and >1.5 (group 3). No protein bands were recognized in samples from Accra, indicating that non-specific binding of human plasma to blackfly salivary antigens is unlikely. In contrast, samples from the endemic Bono East region recognized several protein bands with different intensities. People with an SOD value >0.5 recognized several proteins in the range of 20–40 kDa. Most individuals recognized a protein with an apparent molecular weight (MW) of 17 kDa, two proteins with an approximate MW of 38 kDa, and two proteins with MWs around 60–70 kDa. One major band around 15 kDa was recognized by all samples. The intensity of protein recognition decreased with decreasing SOD values and, as expected, individuals with lower SOD values in the ELISA recognized less protein bands. Interestingly, five samples in the group with an SOD value <0.5, classified as negative according to the ELISA cut-off value, showed strong reactivity with some major protein salivary bands around 15 and 38 kDa, and lower reactivity with bands around 17 and 60–70 kDa.

### Liquid chromatography-tandem mass spectrometry (LC-MS/MS) analysis

**Fig 3:**
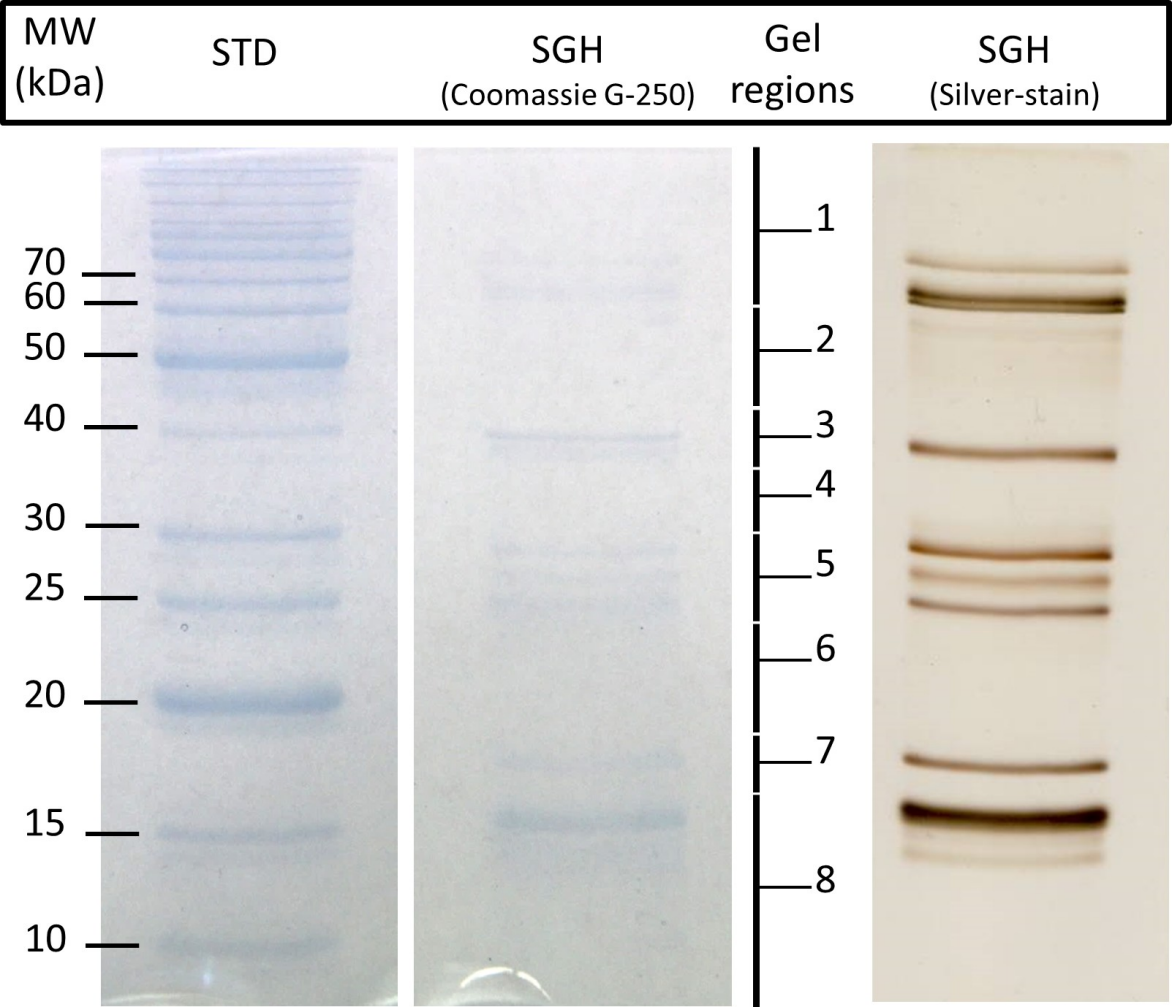
Coomassie G-250 and silver-stained SDS-PAGE gel (12% polyacrylamide). The *Simulium damnosum* s.l. salivary proteins were separated under non-reducing conditions together with a standard (STD) and stained by Coomassie G-250 and the silver-stain method. The separated blackfly salivary gland proteins range from 10 to 80 kDa. Eight different regions were cut from the Coomasssie G-250-stained gel and subjected to mass spectrometry analysis, indicated by ‘Gel regions’. MW: molecular weight; kDa: kiloDaltons; STD: Standard; SGH: Salivary Gland Homogenate.

Based on the results from the immunoblot, several parts of a Coomassie G-250-stained SDS-PAGE gel were cut and analyzed by LC-MS/MS. The different regions that were cut from the gel are indicated on the Coomassie G-250-stained gel in Fig 3, showing that the separated proteins of *S*. *damnosum* s.l. saliva ranged between 10 and 80 kDa. A silver-stained gel was added to better visualize the separated proteins.

Proteins identified by LC-MS/MS are summarized in Table 1. Antigen 5-related proteins and major insect-specific and *Simulium*-specific protein families, enzymes, and protease inhibitors were detected. Antigens with a low MW were not specifically identifiable by mass-spectrometry (gel region 6–8). Apyrase/nucleotidase had the highest intensity in gel regions 1 and 3, salivary hyaluronidase in gel region 2, and salivary secreted antigen 5-related protein in gel regions 4 and 5.

**Table 1:** Proteome analysis of *S*. *damnosum* s.l. salivary proteins. Protein bands of interest were cut from a Coomassie G-250-stained SDS-PAGE gel and analyzed by liquid chromatography-tandem mass spectrometry (LC-MS/MS). The obtained data were screened against available cDNA libraries of Simuliidae, *Culicoides nubeculosus*, *and Culicoides sonorensis*. The identified proteins per gel region are listed according to their intensity with their GenBank accession number, the name of the protein, the species, and its molecular weight. Identified proteins highlighted in bold are the most intensely present per gel region.

Gel region	Matched peptides	Accession number	Protein name	Species	MW (kDa)	Intensity (x 1000)	Database
1 (> 60kDa)	5	AEB96567.1	**Putative apyrase/nucleotidase, partial**	***S*. *guianense* s.l.**	**34.339**	**702620**	**Simuliidae**
	8	AEB96554.1	Salivary alpha-amylase, partial	*S*. *guianense* s.l.	61.471	177750	Simuliidae
	4	ACH56864.1	Salivary alpha-amylase, partial	*S*. *vittatum*	52.206	38972	Simuliidae
2 (45–60 kDa)	1	AEB96520.1	**Salivary hyaluronidase**	***S*. *guianense* s.l.**	**40.217**	**14988**	**Simuliidae**
	1	ACZ28347.1	Putative apyrase/nucleotidase, partial	*S*. *nigrimanum*	54.041	14973	Simuliidae
	4	AEB96567.1	Putative apyrase/nucleotidase, partial	*S*. *guianense* s.l.	34.339	8517.3	Simuliidae
	3	AEB96562.1; ACZ28191.1; ACZ28190.1	Salivary alpha-amylase	*S*. *guianense* s.l., *S*. *nigrimanum*	46.021	2449.8	Simuliidae
3 (35–45 kDa)	3 3	AEB96567.1 ACZ28347.1; ACH56868.1	**Putative apyrase/nucleotidase, partial** Putative apyrase/nucleotidase, partial	***S*. *guianense* s.l.** *S*. *nigrimanum*, *S*. *vittatum*	**34.339** 54.041	**2613.7** 2359.4	**Simuliidae** Simuliidae
4 (30–35 kDa)	1	ACH56844.1; ACH56843.1; ACZ28180.1; ACZ28179.1	**Salivary secreted antigen-5 related protein**	***S*. *vittatum; S*. *nigrimanum***	**32.031**	**10754**	**Simuliidae**
	2	AEB96567.1; ACH56868.1; ACZ28347.1	Putative apyrase/nucleotidase, partial	*S*. *guianense* s.l., *S*. *vittatum; S*. *nigrimanum*	34.339	6338.1	Simuliidae
5 (24–35 kDa)	1	ACH56844.1; ACH56843.1; ACZ28180.1; ACZ28179.1	**Salivary secreted antigen-5 related protein**	***S*. *vittatum*, *S*. *nigrimanum***	**32.031**	**440710**	**Simuliidae**
	1	ACZ28347.1	Putative apyrase-nucleotidase, partial	*S*. *nigrimanum*	34.339	5689.3	Simuliidae

### Molecular identification of blackflies

Sequences obtained from the 18 analyzed simuliid specimens showed an identity of 99.5–100% with haplotypes of the *S*. *damnosum* complex [45], demonstrating that they all belong to *S*. *damnosum* s.l.

## Discussion

### Implications for measuring host exposure to simuliid bites

Studies of hosts bitten by bloodsucking arthropods have demonstrated a specific quantifiable humoral immune response that can potentially serve as an estimate of host exposure to bites of bloodsucking arthropods [16, 18–21]. Since no such assay existed for blackflies, we developed for the first time a human exposure assay for the important onchocerciasis vector group *S*. *damnosum* s.l., allowing us to examine humoral immune responses to salivary proteins by human populations in onchocerciasis (savannah) endemic areas in Ghana. We showed that specific anti-*S*. *damnosum* s.l. IgG and IgM responses are detectable and quantifiable, and we investigated the salivary antigenic proteins associated with these responses.

Previous studies measuring host exposure to salivary proteins of haematophagous arthropods mainly focused on the IgG response. Although IgG is an antibody that develops later after the initial antigen exposure, it has been shown to fluctuate throughout the season in case of sand flies and mosquitoes [46, 47]. This quality renders it ideal as an epidemiological tool to measure biting exposure in host populations. IgM responses, on the other hand, are the first to appear in response to an antigen, and with respect to arthropod saliva, have only been investigated as potential exposure markers of mosquito and triatomine bites [48, 49]. The early rise in specific IgM immune response makes it a potential candidate to evaluate the impact on bite exposure of recently implemented vector control methods, at both the population and individual level. Both IgG and IgM SOD values were substantially and statistically significantly higher in individuals residing in the Bono East region of Ghana than in their negative control counterparts, residing in blackfly-free areas of Ghana (Accra), supporting the notion that people living in onchocerciasis-endemic communities are exposed to *S*. *damnosum* s.l. bites. Although the Bono East region of Ghana has been part of Phase 1 (initiated in 1975) or the South-Eastern extension (started in 1986) of the OCP [30], larvicidal operations ended with the programme closure and fly densities returned to pre-control levels [29]. The lower IgM antibody responses might be indicative of its shorter half-life relative to that of IgG immune responses; however, blackfly biting occurs year-round [37], and the sampling was comprehensive across all age groups (5–95 years).

To further validate the IgG ELISA, samples from people living in blackfly-free rural communities in Gedaref state and in the city of Khartoum, Sudan, were tested showing similarly low and not significantly different baseline antibody levels to the Ghana control group, thus supporting the adopted cut-off values. Due to limitations in blackfly salivary gland availability, the Sudanese samples were not tested with the IgM ELISA. Notably, the antibody responses in the negative control group (in Ghana) of the proposed IgM ELISA were raised, resulting in a higher cut-off value. This may reflect a lower specificity of the IgM ELISA; skin hypersensitivity (IgE-mediated) cross-reactions between salivary allergens of *S*. *vittatum* and *C*. *nubeculosus* have been observed [50], but in sand flies and mosquitoes previous studies only reported cross-reactions between salivary proteins of closely-related species [21, 51, 52]. Evaluation of potential immunological cross-reactions between salivary proteins of *S*. *damnosum* s.l. and other bloodsucking arthropods would be informative to truly identify human exposure biomarkers unique for *S*. *damnosum* s.l..

### Implications for unravelling the salivary proteome of *Simulium damnosum* s.l.

To date, the sialotranscriptomes of only three blackfly species have been unravelled. The first research group to uncover the sialotranscriptome of a member of the Simuliidae family was that of Andersen et al. [53], identifying several anti-haemostatic factors and immunomodulatory activities in *S*. *vittatum* saliva. Subsequent studies reported the sialotranscriptome of two other blackfly species, *S*. *nigrimanum* and *S*. *guianense* s.l. (the latter being a major onchocerciasis vector in the extant Amazonian focus straddling between Venezuela and Brazil [54]), contributing to the identification of additional compounds with potential anti-haemostatic or immunity-related functions [55, 56].

In the present study, antibodies of the Bono East residents recognized numerous *S*. *damnosum* s.l. salivary proteins. Importantly, people residing in Accra and who reported never to have travelled outside of the city, showed no evidence of any cross-reaction with *S*. *damnosum* s.l. salivary proteins, despite them undoubtedly having experienced bites from other haematophagous arthropods, including *Anopheles* mosquitoes [57]. People with lower SOD values reacted to fewer proteins and with lower intensity than people with higher SOD values. All five samples in the group with SOD values of less than 0.5 were classified as ELISA-negative yet showed strong reactivity with one protein band around 15 kDa; two of these five samples also reacted strongly with one band around 38 kDa, and (with a lower intensity) with a protein band around 17 kDa and two bands around 60–70 kDa. This finding could either indicate that the antibody response against these proteins is long-lived, making them a useful marker for past exposure, or that they are just more antigenic and thus potentially the best candidates to measure current exposure to *S*. *damnosum* s.l. bites.

Given the high immunogenicity of the *S*. *damnosum* s.l. salivary proteins, we attempted to characterize these proteins using mass-spectrometry analysis. Protein bands from *S*. *damnosum* s.l. saliva separated by an SDS-PAGE Coomassie G-250-stained gel were thus subjected to LC-MS/MS and results were screened against the available cDNA libraries of Simullidae and of *C*. *nubeculosus* and *C*. *sonorensis*, as biting midges and blackflies are closely related and cross-reactions between salivary allergens of *S*. *vittatum* and *C*. *nubeculosus* have been observed [50]. Several well-known salivary proteins with known anti-haemostatic or immunity-related functions were found in the salivary gland proteome of *S*. *damnosum* s.l., including antigen 5-related protein, apyrase/ nucleotidase, and hyaluronidase. The strong antigens located at 15 kDa and around 17 kDa did not yield any major hits.

Antigen 5-related proteins are associated with IgE hypersensitivity responses against both *S*. *vittatum* and *S*. *nigrogilvum* [25, 58], with the *S*. *vittatum* protein sharing common IgE-binding epitopes with antigen 5-related proteins from *Culicoides nubeculosus* [50]. Apyrases/nucleotidases are ubiquitously found in saliva of haematophagous arthropods. Although the origin of blackfly salivary apyrases has not yet been revealed, sialome studies of *S*. *vitattum*, *S*. *guianense* s.l., and *S*. *nigrimanum* all described transcripts coding for members of the 5’-nucleotidase family, similar to mosquito apyrases [53, 55, 56]. Interestingly, in blackflies apyrase expression was shown to be positively associated with anthropophagy and onchocerciasis vector status of the blackfly species [59]; a trend also observed for *Anopheles* vectors of malaria [60]. Allergenic and antigenic properties have been repeatedly reported for apyrases in mosquitoes and sand flies (reviewed in [61]). Lastly, hyaluronidase has mainly been demonstrated in the saliva of thelmophagous insects (i.e. pool-feeders), such as sand flies, biting midges, tabanid flies, and blackflies [62–65]. By cleaving hyaluronic acid (HA), the enzyme decreases tissue viscosity and consequently helps to enlarge the size of the feeding hematoma and spread other salivary components. It has also been shown to accelerate the bloodfeeding process and to enhance *Leishmania* transmission [65]. Similarly, it has been postulated that hyaluronidase may facilitate transmission of arboviruses of blackflies through co-feeding and by modulating the host immune response [64, 66].

Even though the proteomic analysis included in the present study did reveal the presence of the certain well-known salivary proteins, proper identification of the proteins that were only immunogenic in ELISA-positive individuals appeared challenging with the currently available sialotranscriptomes. Furthermore, the identified proteins overall had a low number of matching peptides, which impedes drawing clear conclusions on immunogen characterization. This work thus highlights the importance of unravelling the sialotranscriptome of this important blackfly species as *S*. *damnosum* s.l. homologues of these and other salivary proteins might be important antigens.

### Conclusions and further research avenues

Quantifying vector biting rates as well as measuring inter-individual variation in host exposure to vector bites, are crucial for parameterizing onchocerciasis transmission dynamics models and predicting the epidemiological impact of control and elimination interventions [6, 7]. The currently used *S*. *damnosum* s.l. HLC studies are usually conducted with a limited team of vector collectors who are positioned in few places known to have high fly densities, resulting in ABR values that are strongly biased towards measuring maximal biting rates rather than true exposure to vector bites. Both the IgG and IgM ELISA proposed in the present study allow for the measurement of anti-vector saliva antibody responses in humans and might be used to complement these *S*. *damnosum* s.l. HLC studies. Furthermore, with the exception of very few observational studies [11], inter-individual variation in exposure has been modelled by applying a suitable statistical distribution to account for heterogeneous biting [7, 9]. Hence, quantifying the individuals’ responses to simuliid salivary antigens and investigating its patterns with host age and sex would greatly assist data-driven calibration of onchocerciasis transmission models [7, 8].

Studies of mosquitoes and sand flies have shown clear seasonal fluctuations in anti-saliva antibody response as well as interpretable correlations with host exposure [15]. Since sampling for the present study was conducted during the (presumably) high biting season (August, wet season), further studies are now needed to evaluate the kinetics and longevity of responses with changes in biting rates (as a result of seasonal fluctuations [46] and anti-vectorial measures [13], and to investigate potential cross-reactions with other bloodsucking arthropods (such as biting midges, mosquitoes, and tabanids) in onchocerciasis-endemic areas. The proteomic results presented in this study provide valuable albeit preliminary information on *S*. *damnosum* s.l. salivary proteins. Further studies on the sialotranscriptome of *S*. *damnosum* s.l. are now needed to fully characterize the most immunogenic and specific proteins, which would ideally result in the production of recombinant antigens that can be employed in onchocerciasis epidemiological and vector control studies.

## Supporting information

S1 File Detailed description of assay optimization and additional results. Text A–Details of the IgG and IgM optimization process. Fig A–Optical density (OD) values for the preliminary immunoassays. Fig B–Distribution of optical density (OD) values for optimization process of immunoassays. Fig C–Comparison of two negative control groups for validation of final IgG ELISA. Table A–Summary statistics of preliminary (OD) and final (SOD) IgG and IgM ELISA.
(DOCX)
