# Supplementary material for: Human immune response against salivary antigens of Simulium damnosum s.l.: A new epidemiological marker for exposure to blackfly bites in onchocerciasis endemic areas
Source: PLoS Negl Trop Dis. 2021 Jun 22;15(6):e0009512. doi: 10.1371/journal.pntd.0009512 (PMC8253393; doi:10.1371/journal.pntd.0009512)
Supplement: S1 File — Text A–Details of the IgG and IgM optimization process. Fig A–Optical density (OD) values for the preliminary immunoassays. Fig B–Distribution of optical density (OD) values for optimization process of immunoassays. Fig C–Comparison of two negative control groups for validation of final IgG ELISA. Table A–Summary statistics of preliminary (OD) and final (SOD) IgG and IgM ELISA. (DOCX) [file pntd.0009512.s001.docx]

# **S1 File - Supporting Information**

# **Human immune response against salivary antigens of *Simulium damnosum* s.l.: a new epidemiological marker for exposure to blackfly bites in onchocerciasis endemic areas**

Laura Willen^1*^, Maria-Gloria Basáñez^2^, Vit Dvorak^1^, Francis B. D. Veriegh^3^, Frank T. Aboagye^3^, Bright Idun^3^, Maha Osman^4^, Mike Y. Osei-Atweneboana^3^, Orin Courtenay^5^, Petr Volf^1^

^1^ Department of Parasitology, Faculty of Science, Charles University, Prague, Czech Republic

^2^ MRC Centre for Global Infectious Disease Analysis and London Centre for Neglected Tropical Disease Research, Department of Infectious Disease Epidemiology, School of Public Health, Imperial College London, London, UK

^3^ Biomedical and Public Health Research Unit, CSIR-Water Research Institute, Accra, Ghana

^4^ Commission for Biotechnology and Genetic Engineering, National Centre for Research, Khartoum, Sudan

^5^ Zeeman Institute for Systems Biology & Infectious Disease Epidemiology Research and School of Life Sciences, University of Warwick, Coventry, UK.

* Corresponding author

E-mail: [laura.willen@gmail.com](mailto:laura.willen@gmail.com)

# **Text A – Details of the IgG and IgM optimization process**

The optimal dilutions for the blackfly salivary gland homogenate (SGH), the anti-human IgG and IgM conjugates and the plasma samples were determined during preliminary assays. For the IgG ELISA, plasma samples from two vector collectors, one randomly chosen sample from the Bono East study area, and three samples from non-simuliid bitten negative controls from Accra were tested at combinations of four plasma dilutions (1/20, 1/50, 1/100, and 1/200) and three antigen concentrations (25 ng, 50 ng and 200 ng per well). For the IgM ELISA, three high responders in the IgG assay were used as positive controls and tested together with two non-simuliid bitten negative controls at combinations of three plasma dilutions (1/20, 1/50, and 1/100) and two antigen concentrations (25 ng and 50 ng per well) (Fig A in S1 File).

Based on the initial results, a larger sample was tested for anti-*Simulium damnosum* s.l. IgG antibodies at antigen concentrations of 50 ng (IgG setup 1) and 200 ng (IgG setup 2) SGH per well, with the sera dilution set at 1/100. The samples chosen for this second assay included 20 negative control samples from people residing in Accra who never left the city and 201 samples of people living in the Bono East Region, Ghana. Similarly, 40 samples were tested for anti-*S. damnosum* s.l. IgM antibodies at sera dilutions 1/50 (IgM setup 1) and 1/100 (IgM setup 2), with an antigen concentration of 25 ng SGH per well. The samples tested consisted of 20 non-simuliid bitten control samples from people residing in Accra and 20 samples of people living in the Bono East Region. The OD values were adjusted for the blank control well (Fig B in S1 File).

In both setups 1 and 2 of the IgG ELISA, a statistically significant difference was obtained between the median of the negative control sera and the median of people residing in the Bono East region (*P*<0.001). Also, a significant difference was found between the medians of the Bono East residents of both setups (*P*<0.001), and between the medians of the negative control samples of both setups (*P*<0.05). The setup with 200 ng of salivary proteins per well was selected for the final development of the assay because this concentration maximized the difference between the negative controls and study area samples (Figs A and B in S1 File). An overview of the descriptive statistics results indicating the mean, median, standard deviation (SD), and interquartile range (IQR) for the optical density (OD) values of the preliminary IgG ELISA is shown in Table A in S1 File.

The IgM ELISA also showed a statistically significant difference between the medians of the negative control sera and the medians of people residing in the Bono East region (*P*<0.001) and between the median OD values of the Bono East residents of the two different setups of the IgM assay (*P*<0.001). No statistically significant difference was found between the negative control samples of both setups (*P*=0.516). Following these observations and keeping the assumption that the study area has a high blackfly biting rate (see *Methods*), the setup with the plasma dilution set at 1/50 was chosen for the final assay. An overview of the descriptive statistics results indicating the mean, median, SD, and IQR for the OD values of the preliminary IgM ELISA is shown in Table A in S1 File.

**Fig A – Optical density (OD) values for the preliminary immunoassays.**
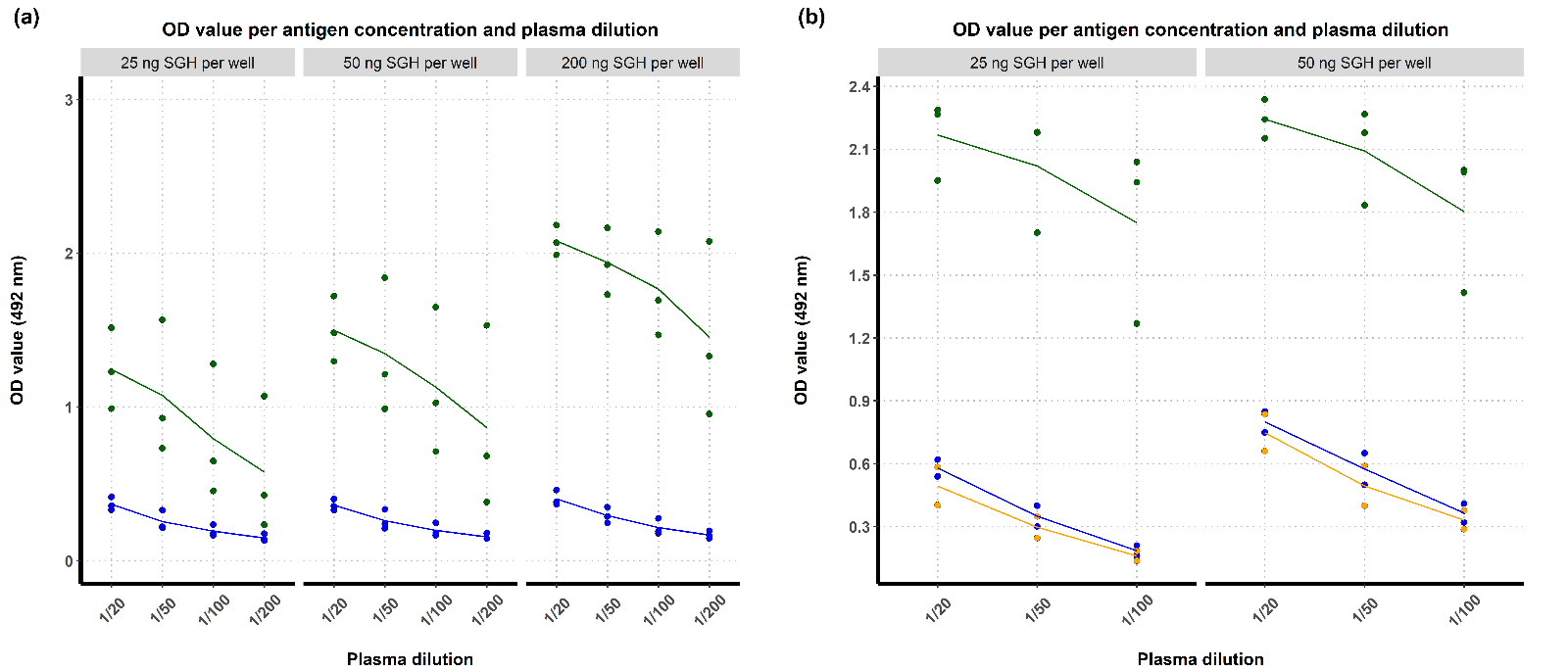
 **(a)** IgG ELISA tested at four different plasma dilutions and three different antigen concentrations per well. Samples from two vector collectors plus one sample from an individual (randomly selected) living in the Bono East region study area (for a total of three samples) are indicated as green solid circles; two negative control samples from people living in the city of Accra, Ghana, are highlighted as blue solid circles. **(b)** IgM ELISA for three different plasma dilutions tested at two different antigen concentrations per well. Samples from two vector collectors are highlighted as orange solid circles; samples from three individuals living in the study area are indicated as green solid circles, and two negative control samples from people living in Accra are highlighted as blue solid circles. The solid lines in corresponding colours are the arithmetic means. OD: Optical Density; ng: nanogram; SGH: Salivary Gland Homogenate.

**
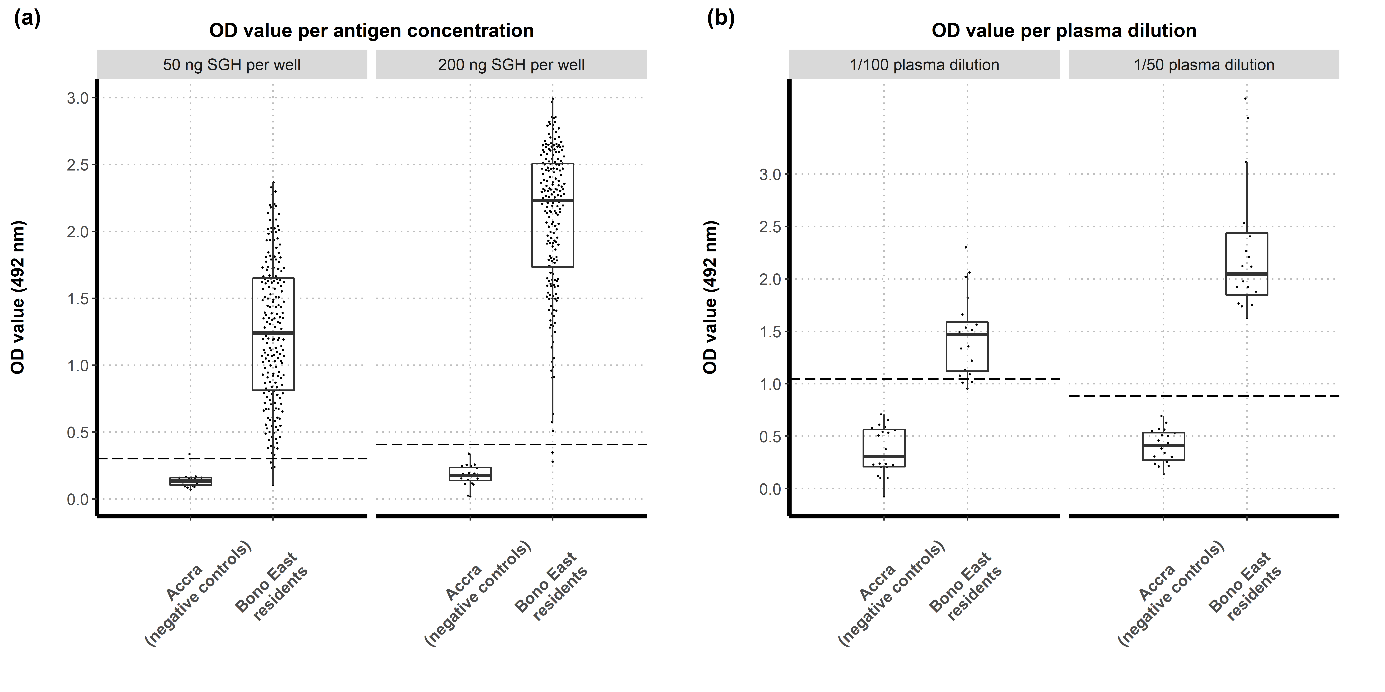
Fig B – Distribution of optical density (OD) values for optimization process of immunoassays. (a)** Box plots of IgG ELISA OD values for negative controls living in Accra (n = 20) and residents of onchocerciasis-endemic areas in the Bono East region, Ghana (n = 201), tested at two different antigen concentrations (50 ng vs. 200 ng SGH) per well. Cut-offs, indicated by the dashed horizontal line, are 0.302 OD and 0.407 OD for the assay with 50 ng SGH and 200 ng SGH per well, respectively. **(b)** Box plots of IgM ELISA OD values for negative controls living in Accra (n = 20) and residents in the Bono East region (n = 20), Ghana, tested at two different plasma dilutions (1/100 vs. 1/50). Cut-offs, indicated with a dashed horizontal line, are 1.046 OD and 0.883 OD for the assay tested at 1/100 and 1/50 plasma dilution, respectively. The solid black horizontal line within the boxes is the median; the lower and upper borders are, respectively, the 1st (Q1) and 3rd (Q3) quartiles; the vertical bars indicate the ‘minimum’ and ‘maximum’ values, calculated as Q1 – 1.5 × IQR (interquartile range) and Q1 + 1.5 × IQR, respectively. OD: Optical Density; ng: nanogram; nm: nanometers; SGH: Salivary Gland Homogenate.

**Fig C – Comparison of two negative control groups for validation of final IgG ELISA.** Box plots of standardized optical density (SOD) values for two negative control groups from Accra, Ghana (n = 35) and Gedaref and Khartoum, Sudan (n = 20). The solid black horizontal line within the boxes is the median; the lower and upper borders are, respectively, the Q1 and Q3 quartiles; the vertical bars indicate the ‘minimum’ and ‘maximum’ values, calculated as Q1 – 1.5 × IQR and Q1 + 1.5 × IQR, respectively. There was no statistically significant difference between the two groups (*P*=0.834). SOD: standardized optical density; nm: nanometers.


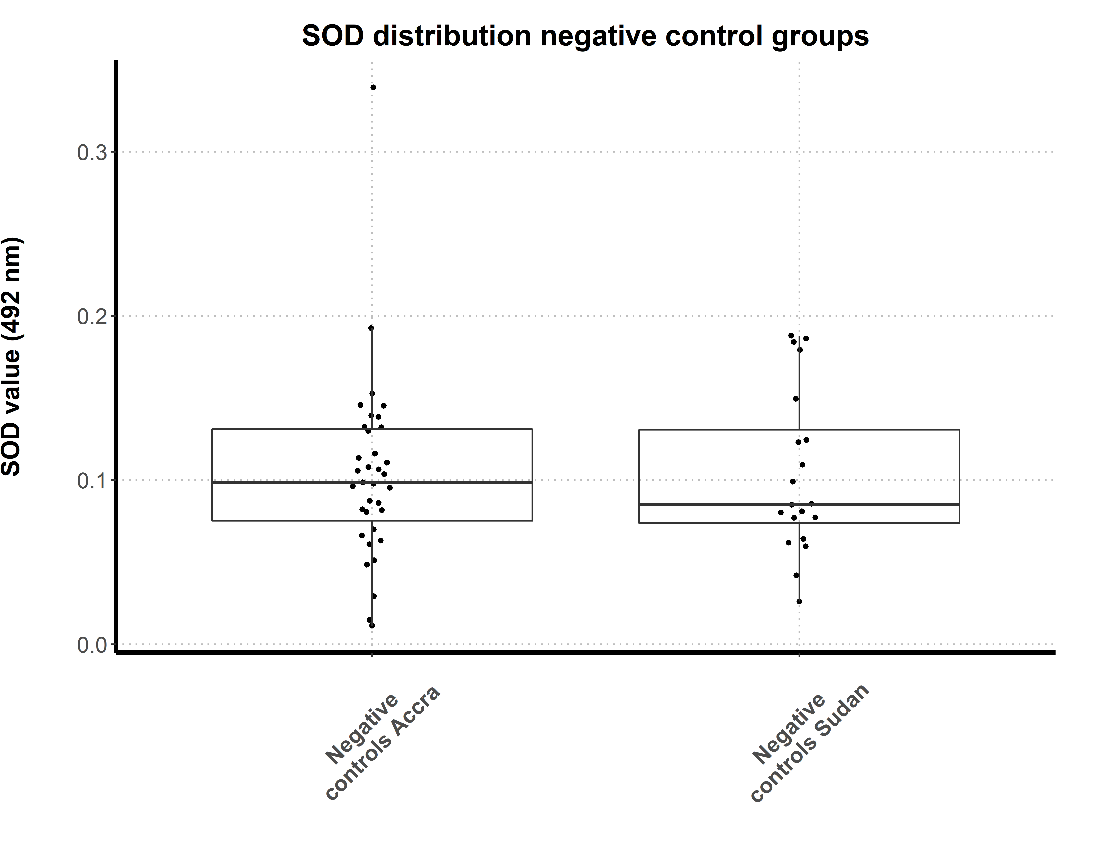


**Table A – Summary statistics of preliminary (OD) and final (SOD) IgG and IgM ELISA.** Arithmetic mean, median, standard deviation (SD), interquartile range (IQR) and cut-off values are shown per assay. **(a)** results from the IgG ELISA; **(b)** results from the IgM ELISA.

| (a) | Antigen concentration per well | Area | n | Mean | SD | Median | IQR | Cut-off |
| --- | --- | --- | --- | --- | --- | --- | --- | --- |
| Preliminary IgG assay | 50 ng SGH | Accra  (negative controls) | 20 | 0.139 | 0.0546 | 0.134 | 0.0549 | 0.302 |
|  |  | Bono East residents | 201 | 1.25 | 0.535 | 1.24 | 0.838 |  |
|  | 200 ng SGH | Accra  (negative controls) | 20 | 0.175 | 0.0775 | 0.177 | 0.0979 | 0.407 |
|  |  | Bono East residents | 201 | 2.09 | 0.545 | 2.23 | 0.773 |  |
| Final IgG assay | 200 ng SGH | Accra  (negative controls) | 35 | 0.104 | 0.0570 | 0.0987 | 0.0557 | 0.275 |
|  |  | Bono East residents | 958 | 1.29 | 0.292 | 1.35 | 0.355 |  |

| (b) | Plasma dilution | Area | n | Mean | SD | Median | IQR | Cut-off |
| --- | --- | --- | --- | --- | --- | --- | --- | --- |
| Preliminary IgM assay | 1/50 | Accra  (negative controls) | 20 | 0.405 | 0.159 | 0.410 | 0.263 | 0.883 |
|  |  | Bono East residents | 20 | 2.25 | 0.601 | 2.05 | 0.589 |  |
|  | 1/100 | Accra  (negative controls) | 20 | 0.362 | 0.228 | 0.307 | 0.354 | 1.046 |
|  |  | Bono East residents | 20 | 1.45 | 0.377 | 1.47 | 0.468 |  |
| Final IgM assay | 1/50 | Accra  (negative controls) | 33 | 0.416 | 0.194 | 0.384 | 0.252 | 0.998 |
|  |  | Bono East residents | 500 | 1.13 | 0.594 | 0.975 | 0.607 |  |

SGH: Salivary Gland Homogenate; n: number of samples; SD: Standard deviation;
IQR: Interquartile Range; OD: optical density (used for the preliminary assays);
SOD: standardized optical density (used for the final assays); ng: nanograms.
